# Assessing vestibular function in patients with vestibular schwannoma: a comprehensive multi-test vestibular evaluation

**DOI:** 10.1007/s00405-025-09691-4

**Published:** 2025-11-14

**Authors:** Francesco Comacchio, Valerio Maria Di Pasquale Fiasca, Giovanni Poli, Giulia Tealdo, Barbara Bellemo, Paola Magnavita, Giulia Zattoni, Elisabetta Zanoletti

**Affiliations:** 1 Otolaryngology Unit and Regional Specialised Centre for diagnosis and treatment of Vertigo, Sant’Antonio Hospital, Via J. Facciolati 71, 35127 Padua, Italy; 2 University of Padua, Department of Neuroscience, Via Giustiniani 2, 35128 Padua, Italy; 3 University Hospital of Padua, Unit of Otorhinolaryngology, Via Giustiniani 2, 35128 Padua, Italy

**Keywords:** Vestibular schwannoma, Vestibular disease, Vestibular test, Caloric test, Video head impulse test, Vestibular evoked myogenic potential

## Abstract

**Purpose:**

We present the results of a battery of vestibular tests on a cohort of Vestibular Schwannoma (VS) patients. We aim to describe the efficacy and sensitivity of those tests in assessing vestibular function and identifying impairment caused by VS.

**Methods:**

A retrospective study was conducted in a tertiary referral centre, the University Hospital of Padova and the Regional Specialised Centre of Veneto Region for Diagnosis and Cure of Vertigo (Sant’Antonio University Hospital). We enrolled and evaluated 50 patients referred for surgical treatment and newly diagnosed with undergoing observational management. The patients underwent a vestibular multi-tests in-home protocol, including videonystagmography (VNG), caloric tests (CalT), video head impulse test (vHIT), cervical and ocular vestibular evoked myogenic potentials (cVEMPs and oVEMPs) and posturography. We analysed the alteration rate of vestibular tests in detecting vestibular impairment caused by VS.

**Results:**

Vestibular tests showed high levels of sensitivity, which increased in the case of simultaneous use (VNG and CalT with vHIT: 100%). The results of vHIT tests also correlated with tumour characteristics such as size and location (*p* < 0.05).

**Conclusions:**

We comment on the usefulness of a multi-test vestibular evaluation of VS patients. Integrating these tests improves the sensitivity of detection of vestibular impairment.

## Introduction

Vestibular schwannoma (VS) is a tumour that develops from Schwann cells of the VIII cranial nerve. It most frequently originates from the vestibular branch and occurs sporadically and unilaterally [1], leading to hearing loss and vestibular symptoms such as vertigo, instability and dizziness [2], with significant variability [3]. In advanced stages, the increasing size of the tumour may result in compression of the brainstem, cerebellum and ventricles, contributing to instability. Vestibular symptoms are known to be less frequent, auditory impairments being the most frequent [4], but contribute to the development of anxiety and depression [5] with significant impact on the quality of life [6].

In small tumours, the current therapy of Vs has shifted the focus to functional outcomes, particularly concerning facial nerve and hearing function [7]. Observation protocols and active therapies (radiotherapy and surgery) have been proposed with different outcomes. Early surgery on small tumours, for preservation or rehabilitation of facial and auditory function, is becoming a viable option, especially in growing tumours [8]. Conversely, the impact of VS on the balance system and the potential for clinicians to address this aspect of the illness remains an open question. Balance function relies on the complex interactions between vision, proprioception and the vestibular system [9]. Physiological compensation after loss of vestibular function, depending on the contralateral vestibular organ with central integration of vestibular, ocular and proprioceptive inputs, has generally been considered sufficient to recover from this handicap. The resulting vestibular impairment has often been disregarded. As available today with new diagnostic tools, assessment of balance impairment helps predict the risk of vestibular decompensation after treatment [10], identifying patients likely to benefit from vestibular rehabilitation. It can be applied in the follow-up setting over the wait-and-scan protocols and pre-surgical and post-surgical phases [3].

Specific tests like videonystagmography (VNG), caloric tests (CalT) [11], video head impulse test (vHIT) [12], cervical and ocular vestibular evoked myogenic potentials (cVEMPs and oVEMPs) [13, 14] and posturography [15] are administered to assess residual function of the vestibular organ and detect VS-related vestibular impairment [14, 16, 17]. The accuracy of these vestibular exams in diagnosing VS remains under study. In the present study, we analysed the results of a battery of vestibular tests in a non-consecutive cohort of VS-affected patients. Our aim was (i) to describe the alteration rate of a series of vestibular tests in VS patients; (ii) analyse the relationship between vestibular function and VS size.

## Materials and methods

This retrospective study retrieved data from a prospectively maintained database in a cohort of patients affected by sporadic VS. Diagnosis was obtained with contrast-enhanced cerebellopontine angle (CPA) Magnetic Resonance Imaging (MRI). Patients were first evaluated in the Otoneurosurgical-Skull base Centre of the University Hospital of Padova and submitted to a specific in-home protocol within the Regional Specialised Centre of Veneto Region for Diagnosis and Cure of Vertigo at the St. Antonio Hospital of Padova, from October 2022 to September 2024. Patients were included according to these selection criteria:

- Patients referred for surgical treatment who preliminarily underwent an established preoperative vestibular assessment;
- Patients with newly diagnosed vestibular schwannoma (VS) complaining of high levels of vestibular symptoms.

Informed consent was obtained from all subjects involved in the study. General and demographic information such as sex, age and general anamnesis were collected. Data regarding vestibular symptoms and history were retrieved, together with radiological characteristics of the VS (position, size and shape). Tumour dimensions were measured in mm as the maximum size along the tumour’s major axis, in the largest diameter in the CPA, and with the further length in the Internal Acoustic Canal (IAC).

A selected cohort of patients underwent a comprehensive battery of vestibular tests as follows:

- Videonystagmography: assessment of spontaneous nystagmus in central and lateral gaze, positional nystagmus, and oculomotricity (smooth pursuit and saccadic movements) using the ICS Chartr 200 (Natus, Taastrup, Denmark, formerly Otometrics).
- Caloric tests: bilateral bithermic caloric tests (cold at 30 °C and warm at 44 °C) using the ICS Chartr 200 (Natus, Taastrup, Denmark, formerly Otometrics).
- Video Head Impulse Test: evaluation of all semicircular canals (SC) was conducted using ICS Impulse (Natus, Taastrup, Denmark, formerly Otometrics) and EyeSeeCam (Interacoustics VisualEyesTM, Middelfart, Denmark) equipment. A deficit was identified where the absolute Gain was < 0.7 in PSC and ASC, < 0.8 in HSC, or > 0.3 compared to the contralateral side. The presence of significant corrective saccades in the HIMP protocol was also investigated.
- Vestibular Evoked Myogenic Potentials: ocular and cervical vestibular evoked myogenic potentials were conducted via air and bone conduction (AC-cVEMPs and BC-oVEMPs) using the Eclipse (Interacoustic) equipment. cVEMPs were elicited using a 500 Hz tone burst, with a rise-fall time and plateau of 4.0 ms. The tone bursts were presented monaurally through headphones (Telephonics TDH-49) at 70, 90, and 105 dB HL, with a repetition rate of 5.1 bursts/s. EMG activity was amplified and filtered within a bandwidth of 10 to 1500 Hz. At least 200 cVEMPs responses were obtained for each recording and then averaged. The same stimulus, with filtering at 0.1–1000 Hz, was administered via a Radio-Ear B71 bone transducer for oVEMPs recording. Waveforms p1-n1 for cVEMPs and n1-p1 for oVEMPs and their amplitudes were evaluated.
- Posturography: balance conditions in static and dynamic postural activities were assessed using the BASIC Balance Master (NeuroCom Balance Manager, Neurocom Inc, Oregon, USA).

All tests that showed abnormalities in the examinations were reported as positive. The alteration rates of each test in our sample were reported.

Statistical analyses were performed using the R software [18]. In the initial exploratory analyses, nominal variables were represented as counts and frequency percentages (N and %), while quantitative variables were reported as mean and standard deviation (SD) if normally distributed, or as median and interquartile range (IQR) if not normally distributed. For each test, sensitivity data were provided. The gain of the semicircular canals was reported as mean and standard deviation. A correlation analysis was then performed between the size of the VS and the gains of the semicircular canals evaluated with vHIT. Spearman’s rho and Pearson’s r coefficients were applied.

The study was conducted in accordance with the Declaration of Helsinki and approved by the Institutional Ethics Committee of the University Hospital of Padova 468n, 7 March 2024.

## Results

65 patients were enrolled in the study, though the previously established whole battery of tests was completed and available in 50 cases. Eight patients were excluded due to the absence of caloric tests, and 7 due to the inability to complete the tests’ required tasks (e.g., very low visual acuity, previous blind sac ear closure, etc.). The demographic and radiological data of the resulting 50 patients are reported in Table 1.

**Table 1:** Demographic and radiological characteristics (R: right; L: left; F: female; M: male; CPA: cerebellopontine Angle)

Variable	Results
N	50
Side (R: L)	23:27
Sex (F: M)	26:24
Age+	56.92 (10.53)
Vestibular anamnesis*	24 (48%)
CPA size mm+	13.51 (9.78)
Intrameatal/Extrameatal*	22 (44%)/28 (56%)

*N(%); +Mean (SD)

**Fig. 1:**
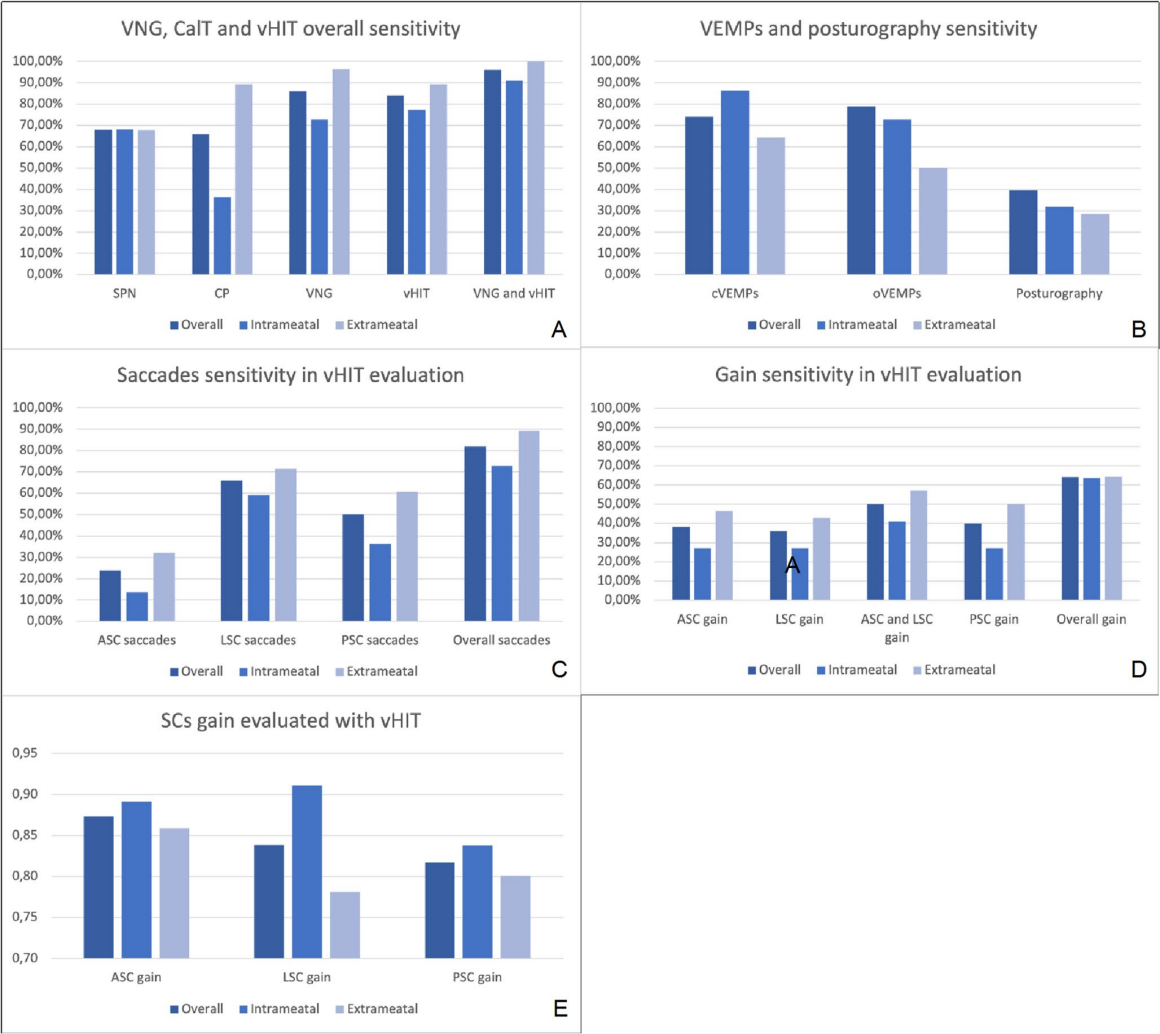
**A**: Sensitivity of Videonystagmography (VNG), Caloric Tests (CalT), and video Head Impulse Test (vHIT) reported overall or divided into intrameatal and extrameatal VS subgroups. **B**: Sensitivity of ocular and cervical Vestibular Evoked Myogenic Potentials (oVEMPs and cVEMPs) an posturography reported overall or divided into intrameatal and extrameatal VS subgroups. **C**: Sensitivity of saccades evaluated with vHIT among the three semicircular canals (SCC), reported overall or divided into intrameatal and extrameatal VS subgroups. **D**: Sensitivity of gain evaluated with vHIT among the three SCC, reported overall or divided into intrameatal and extrameatal VS subgroups. **E**: Gain evaluated with vHIT among the three SCC, reported overall or divided into intrameatal and extrameatal VS subgroups. (ASC: Anterior Semicircular Canal; LSC Lateral Semicircular Canal; PSC: Posterior Semicircular Canal)

The sensitivity rates of vestibular tests in assessing the degree of vestibular impairment are reported in Table 2; Fig. 1.

**Table 2:** Videonystagmography, caloric tests, vHIT saccade and gain and cVEMPs, oVEMPs and posturography sensitivity in VS evaluation. Among vemps, abnormal wave shape (reduced or absent) was considered as a positive evaluation. Bilaterally absent waves were not included in the analysis

	SPN	Caloric paresis	VNG	vHIT	VNG and vHIT	cVEMPS	oVEMPs	Posturography	
Overall*	68.00%	64.00%	86.00%	90.00%	96.00%	74.00%	78.95%	39.47%	
Intrameatal*	68.18%	36.36%	72.73%	90.90%	90.91%	86.36%	72.73%	31.82%	
Extrameatal*	67.86%	85.71%	96.43%	89.20%	100.00%	64.29%	50.00%	28.57%	
	**vHIT Saccades**				**vHIT Gain**				
	**ASC**	**LSC**	**PSC**	**Total**	**ASC**	**LSC**	**ASC and LSC**	**PSC**	**Total**
Overall *	24.00%	66.00%	50.00%	82.00%	38.00%	38.00%	50.00%	40.00%	64.00%
Intrameatal*	13.64%	59.09%	36.36%	72.73%	27.27%	27.27%	40.91%	22.73%	59.09%
Extrameatal*	32.14%	71.43%	60.71%	89.29%	46.43%	46.43%	57.14%	50.00%	64.29%

*N(%)

High alteration rate was achieved by vHIT (90.00%) and VNG (86.00%), especially if coupled (96.00%), with higher rates observed in the extrameatal (100%) than in the purely intrameatal (90,91%) group of VS patients. Combination of the two tests, vHIT and VNG, allowed a complete assessment of all the SCs to be performed, extending the analysis to the vertical SCs. Among the vHIT results, overall analysis of the presence of saccades presence reported an 82.00% sensitivity, with a higher rate in the extrameatal VS group (89.29%), and remarkably higher than what was observed in the gain analysis (64.00% overall, 64.29% extrameatal). The sensitivity of these two tests was the highest reported in our study. Other tests like CalT, cVEMPs and oVEMPs showed varied levels of sensitivity (respectively 64.00%, 74.00%, 78.95%). A lower level was described in the case of the posturography (39.47%).

In addition, the gain analysed by the vHIT in the three SC on the affected side is reported in Table 3; Fig. 1.

**Table 3:** vHIT gain of the three semicircular canals and caloric paresis analysed with CalT

	ASC gain	LSC gain	PSC gain	Caloric paresis
Overall *	0.87 (0.29)	0.84 (0.28)	0.82 (0.26)	51.57 (35.91)
Intrameatal*	0.89 (0.28)	0.91 (0.25)	0.84 (0.26)	28.16 (30.23)
Extrameatal*	0.86 (0.30)	0.78 (0.30)	0.80 (0.26)	67.47 (30.67)

*Mean (SD)

As can be noticed, a mean higher gain was reported in the ASC. This finding agrees with a less frequent presence of saccades, which was described in the same canals. The results of VEMPs evaluation are reported in Table 4.

**Table 4:** vHIT gain of the three semicircular canals and caloric paresis analysed with CalT

	ASC gain	LSC gain	PSC gain	Caloric paresis
Overall *	0.87 (0.29)	0.84 (0.28)	0.82 (0.26)	51.57 (35.91)
Intrameatal+	0.90 (0.35)	0.95 (0.16)	0.83 (0.33)	26 (59)
Extrameatal*	0.86 (0.30)	0.78 (0.30)	0.80 (0.26)	67.47 (30.67)

*Mean (SD); +Median (IQR)

**Fig. 2:**
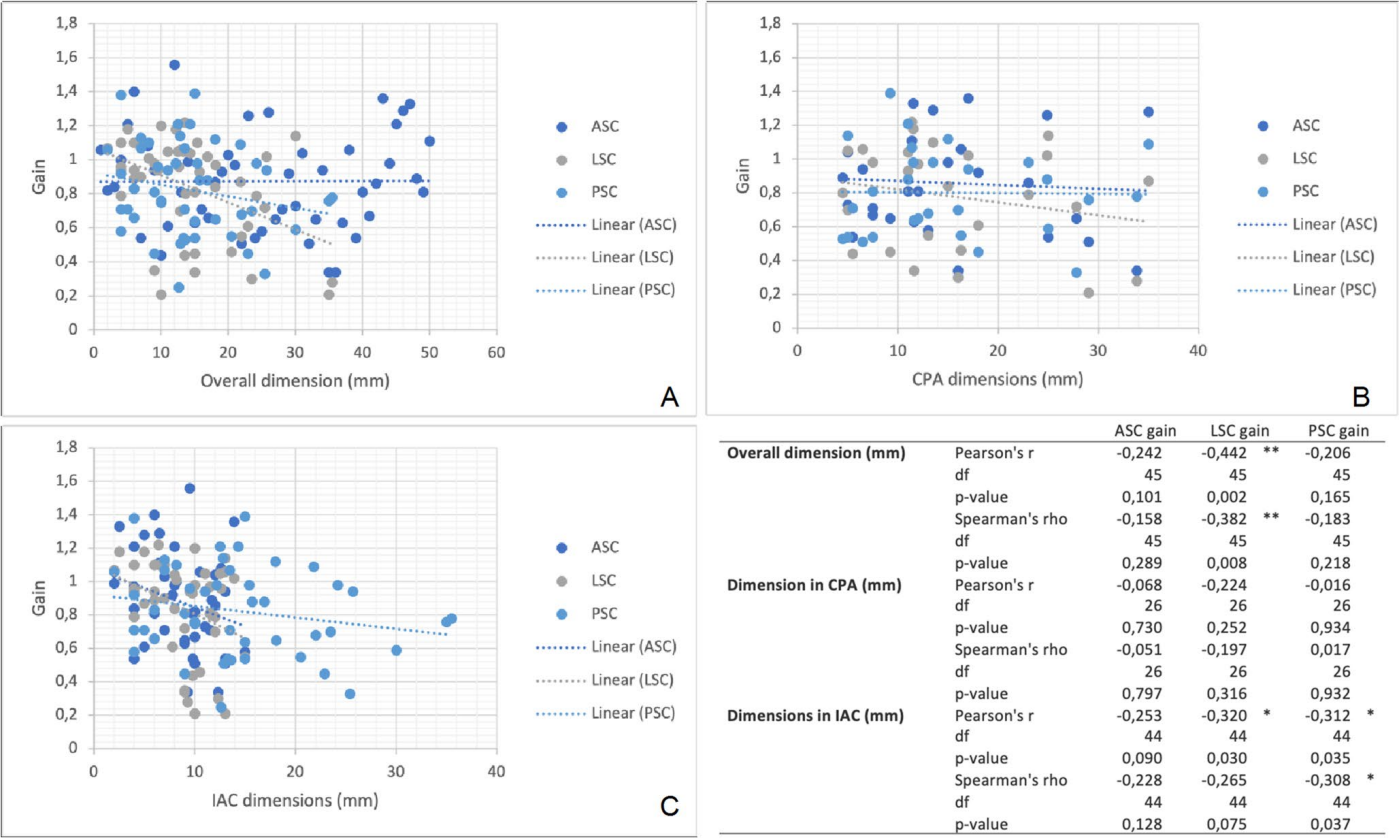
**A**: linear correlation between overall dimensions and gain evaluated with vHIT among the three semicircular canals (SCC). **B**: linear correlation between dimensions inside the Internal Acoustic Canal (IAC) and gain evaluated with vHIT among the three SCC. **C**: linear correlation between dimensions in the Cerebellopontine Angle (CPA) and gain evaluated with vHIT among the three SCC. *p < 0.05 **p < 0.005. (ASC: Anterior Semicircular Canal; LSC Lateral Semicircular Canal; PSC: Posterior Semicircular Canal)

The correlation between tumour dimensions in mm and vHIT gain was tested, as shown in Fig. 2. LSC showed a statistically significant correlation with both IAC (*p* = 0.03) and overall dimensions (*p* = 0.002), while PSC showed a correlation only in IAC dimensions (*p* = 0.035).

## Discussion

VS causes damage to the vestibular function due to its origin from the vestibular branches of the eighth cranial nerve and its effect on vestibular arteries. The entity of vestibular dysfunction and related symptoms are influenced by factors such as tumour size, time elapsed since the onset of the disease, patient’s health, comorbidities and pre-existing or concomitant vestibular impairment conditions. Each type of vestibular test highlights different aspects of the affected balance organ. The VNG provides a general analysis of the function of peripheral and central vestibular pathways. CalT [11] are generally associated with VNG and involve bilateral recording of the function of the lateral semicircular canals. The vHIT [12] allows for the study of all six semicircular canals. VEMPs are used to evaluate the conduction of neural information along the vestibular nerves to the saccular organs: cervical VEMPs [13, 14] assess the inferior vestibular nerve and the saccule, while ocular VEMPs evaluate the superior vestibular nerve and the utricle. Lastly, posturography [15] allows the study of balance function using a stabilometric platform. The use of these tests may prove relevant in the diagnostic setting. A wide range of sensitivities of available tests is reported in the literature [13, 17], which raised the opportunity for a comprehensive battery of multi-tests to be applied in the diagnostic setting and before treatment. Unexplained asymmetry at vestibular tests should prompt imaging in patients presenting with non-specific vestibular symptoms [14].

### VNG

VS can be revealed by spontaneous or positional nystagmus, which was reportedin almost half of VS patients, with higher rates in the cases of larger tumour size [19]. Nystagmus have been described in the literature, such as Brun’s nystagmus in larger VS [20]. Spontaneous nystagmus was detected in 24% [21] of cases among 121 VS patients, mainly those with larger and growing tumours. Our series showed heterogeneous spontaneous or provoked nystagmus without a specific pattern. Our patients were all scanned with VNG, which generally showed vestibular impairment in 68% of patients, with little differences between subgroups of intrameatal and extrameatal tumours (68.18% and 67.86% respectively).

### CalT and vHIT

Two types of tests provide SC evaluation: CalT and vHIT. They both allow the study of the vestibulo-ocular reflex (VOR); the former evaluates the slow frequencies of the reflex, while the latter analyses a faster range of frequencies. These tests should be considered complementary for the functional assessment of SC function and VOR [24].

In the literature, CalT displays a sensitivity ranging from 43 to 90% [15, 17, 22, 23] In our experience, it showed a sensitivity of 64%, remarkably higher in extrameatal VS (85.71%) than in intrameatal (36.36%).

The vHIT enables the evaluation of the SCs vestibular function by recording the VOR’s gain in response to passive head movements in the planes of the three SCs. Following a unilateral vestibular lesion, VOR function is altered, resulting in the need for corrective saccades in the direction of the reflex and a reduction in VOR gain, which are assessed during the test. In this study, a threshold of 0.7 gain for vertical canals and 0.8 for the lateral canal was applied, and the presence of saccades was evaluated. The meaning of borderline results remained uncertain, where pathological saccades were present with normal or low mean gain values without accompanying pathological saccades. Technical limitations, procedural errors in testing, and patients’ low cooperation [2] may have accounted for these results. Jensen et al. [25] applied the vHIT to test vestibular impairment in VS patients, achieving a sensitivity of 61.8%. In addition, these authors stated that the evaluation of saccades, coupled with the gain, resulted in a reduction of diagnosis (32.7%) in the sample, highlighting an overestimation of pathology when relying solely on gain evaluation. Therefore, they recommend examining saccades to improve the accuracy of the examinations. In our experience, the evaluation of saccades had a higher sensitivity (82%) than the evaluation of SC gain (64%).

It is recognised that vHIT should be performed on each VS patient, preoperatively and postoperatively [26]. The sensitivity rate of the vHIT in VS patients ranged from 27 to 90% for lateral canal vHIT [14, 16, 22, 26–28]. The ASC appeared to be the most robust, demonstrating lower rates of altered results in both gain and the presence of saccades. In the literature, LSC and PSC vHIT are more sensitive than ASC vHIT, with reported sensitivities of 27%−57% in the LSC and 8%−36% in the PSC, respectively [2, 14, 16, 27–29]. This may be due to the difficulty of performing the head movement necessary for vertical SC examination, which is lower than that required for LSC testing. Moreover, vertical canal testing is more inter-examiner dependent than lateral testing [30]. In our experience, the general sensitivity of vHIT was reported as 90%, with little difference among different sizes (intrameatal 89.2%, extrameatal 90%). The ASC was the most robust (24% in saccades evaluation and 38% in gain); this result agrees with what was reported in the literature.

Due to the high variability of vestibular impairment, a study of all three canals through vHIT is paramount. The degree of sensitivity of the vHIT compared to CalT is a topic of discussion. A study from 2015 [31] showed that the vHIT demonstrated greater sensitivity in detecting LSC VOR alterations, but West et al. (2020) [17] did not confirm this result, where higher sensitivity was evidenced in CalT.

The combined evaluation of vHIT and VNG with caloric testing could allow the most comprehensive assessment of the vestibular organ in VS patients. Conversely, an evaluation relying solely on VNG and CalT would not allow the study of the PSC, which is innervation from the inferior branch of the vestibular nerve. Another recent study [26] reported that the vHIT was reliable in detecting vestibular impairment in patients commonly appearing on VNG. Nevertheless, exclusive evaluations with the vHIT would miss the findings obtained by the CalT, a test with proven high sensitivity.

### Posturography

Postural control depends on integrating somatosensory, vestibular and visual information. The VS on the vestibular system can produce a unilateral degradation of vestibular function, progressively compensated for by central adaptive mechanisms [31]. Posturography assesses somatosensory, visual and vestibular function in maintaining overall body balance [32]. In our sample, the assessment showed low sensitivity levels (39%) in the vestibular function evaluation of the Sensory Organization Test (eyes closed and foam platform), with little difference between tumours of bigger and smaller sizes. In the literature, posturography has been reported as capable of detecting balance affection produced by VS, and postural unsteadiness was detected in 17%−62% of patients [33], with higher levels in tumours contacting the brainstem.

### Cervical and ocular VEMPs

The analysis of vestibular pathways with VEMPs provides information on the condition of the vestibular nerve and macular organ [34]. VS affects the neural pathway, resulting in prolonged latency [35], reduced amplitude, and changes in wave shape up to the point of being unrecognisable [36]. Ushio et al. [37] reported no significant differences between CalT and VEMPs sensitivity in a group of 803 consecutive patients. Our results showed a sensitivity of 74% for cVEMPs and 78.95% for oVEMPs. A percentage of patients was not assessable with oVEMPs due to the bilateral absence of waveforms, especially in the extrameatal subgroup. These data agree with the literature: in other studies, a sensitivity of 50%−80.8% has been reported for cVEMPs [16, 38, 39] and 50%−76% for oVEMPs [12, 37].

### Factors correlating to the vestibular function in VS patients

Hearing status, tumour size and vestibular symptoms were proposed as multifactorial causes impacting vestibular structures, perhaps related to dysfunction of the SCs [40] and macular organs. The correlation between tumour volume and vestibular function was described in various studies [14, 17, 22, 28, 38–40]. Nilsen et al. [33] reported that the correlation between dimensions and vestibular impairment was also detected in small and medium-sized VS and was evident in all three SCs. Although present, the meaning of this correlation has to be fully clarified [13, 15], as the damaging effect of VS, mainly due to tumour size and position in the IAC, depends either on direct compression of the VIII cranial nerve, or factors secreted by the tumour [41] and damage to the vestibular arteries. In our series tumour size was analysed. LSC gain loss was significantly correlated with larger tumour sizes (*p* < 0.01). This result was not reported in the vertical canals.

### Future perspectives

The attempt to detect the nerve of origin of the tumour (superior or inferior vestibular nerve or both) is a further application of vestibular study. This investigation has garnered scientific interest, as the origin of the VS could correlate with more or less favourable prognoses in observational protocols and postoperative functional outcomes. This probably depends on the different positions the tumour assumes, depending on the area from which it develops. The inferior vestibular nerve, which appears to be the more frequent origin of the tumour, places the tumour closer to the acoustic nerve compared to when the VS originates from the superior branch of the vestibular nerve [42]. This seems to correspond to a lower rate of postoperative hearing preservation in tumours originating from the inferior branch [43]. The results of our research regarding this topic will be the subject of a subsequent publication by our research group.

### Multi-test evaluation

The VS effect on vestibular function is often underestimated in clinical practice. In our study, we comment on the relevance of this aspect and the usefulness of a multi-test vestibular evaluation. This assessment aims to more accurately analyse the vestibular condition of affected patients, as individual tests alone may not capture the full spectrum of vestibular dysfunction caused by VS. Each test provides information on different aspects of the patient’s vestibular function, complementing one another. VS can damage various components of the vestibular nerves, leading to diverse and unpredictable functional effects. Therefore, conducting a vestibular assessment that accounts for this heterogeneity is highly recommended by employing multiple tests. The sensitivity level of the battery test may be satisfactorily high with low costs. As the understanding of vestibular dysfunction in VS evolves, clinicians should incorporate vestibular assessments into VS patients’ care, aiming for more personalised and functionally oriented treatment strategies. Although suggested as an alternative to MRI for patients with asymmetric hearing [17], its promising role in the diagnostic screening of VS has to be fully explored, to assess if it may become a reliable option to investigate unilateral impairment in vestibular-impaired patients before recommending MRI scanning [38]. Furthermore, it must be remarked that vestibular multi-testing does not replace MRI in the diagnostic assessment of VS, but are both useful and complementary. Vestibular assessment allows for a detailed evaluation of function, offering insights into the nature of the vestibular system. In parallel, MRI contributes as a definitive tool for diagnosis, showing the presence or not of VS, and enables the identification of potentially coexisting causes of vestibular and auditory impairment.

## Conclusions

This study underlines that vestibular dysfunction in patients affected by VS is common, heterogeneous, and often underrecognised, with important diagnostic and prognostic implications. Our findings demonstrate that a comprehensive battery of vestibular tests - particularly vHIT and VNG combined with CalT - provides high sensitivity in detecting vestibular impairment, especially when used together. This approach enables a more thorough evaluation of the VOR, including the vertical semicircular canals, thereby improving diagnostic accuracy.

The observed correlation between tumour size and reduced gain in the LSC underscores the role of tumour volume and location in vestibular dysfunction. While cVEMPs and oVEMPs showed variable sensitivity, they offer valuable complementary insights, especially regarding the selective involvement of superior or inferior vestibular nerve branches. Although posturography showed lower sensitivity, it may still contribute to the clinical picture in cases presenting with subjective imbalance.

Our results support the routine integration of a multi-test vestibular assessment in the clinical management of VS patients, both at diagnosis and during pre- and post-operative care. This approach enables a more precise identification of patients at risk of vestibular decompensation, providing critical information for planning personalised treatment and rehabilitation strategies. Given the low cost and high diagnostic value, a vestibular evaluation should be considered a standard component of the workup for VS. Future prospective studies are warranted to explore the diagnostic utility of this battery as a potential diagnostic tool for patients with unilateral, nonspecific vestibular symptoms.

## References

[1] West N, Møller MN, Hansen S, Cayé-Thomasen P (2018) Audiovestibular loss of function correlates in vestibular schwannomas. J Int Adv Otol 14:161–165. 10.5152/iao.2018.5500 30100546 10.5152/iao.2018.5500 PMC6354468

[2] Kjærsgaard JB, Szeremet M, Hougaard DD (2019) Vestibular deficits correlating to dizziness handicap inventory score, hearing loss, and tumor size in a Danish cohort of vestibular Schwannoma patients. Otol Neurotol 40:813–819. 10.1097/MAO.0000000000002236 31135674 10.1097/MAO.0000000000002236

[3] Kentala E, Pyykkö I (2001) Clinical picture of vestibular schwannoma. Auris Nasus Larynx 28:15–22. 10.1016/s0385-8146(00)00093-6 11137358 10.1016/s0385-8146(00)00093-6

[4] Peris-Celda M, Graffeo CS, Perry A et al (2019) Main symptom that led to medical evaluation and diagnosis of vestibular schwannoma and patient-reported tumor size: cross-sectional study in 1,304 patients. J Neurologic Surg Part B: Skull Base 80:316–322. 10.1055/s-0038-1675175 10.1055/s-0038-1675175 PMC6534738 31143577

[5] Chen X, Wei D, Fang F et al (2024) Peripheral vertigo and subsequent risk of depression and anxiety disorders: a prospective cohort study using the U K biobank. BMC Med 22:63. 10.1186/s12916-023-03179-w 38336700 10.1186/s12916-023-03179-w PMC10858592

[6] Chweya CM, Tombers NM, Lohse CM et al (2021) Disease-specific quality of life in vestibular schwannoma: a National cross-sectional study comparing microsurgery, radiosurgery, and observation. Otolaryngol Head Neck Surg 164:639–644. 10.1177/0194599820941012 32689889 10.1177/0194599820941012

[7] Zanoletti E, Mazzoni A, d’Avella D (2019) Hearing preservation in small acoustic neuroma: observation or active therapy? Literature review and institutional experience. Acta Neurochir (Wien) 161:79–83. 10.1007/s00701-018-3739-x 30535851 10.1007/s00701-018-3739-x

[8] Zanoletti E, Concheri S, Tealdo G, Cazzador D, Denaro L, d’Avella D, Mazzoni A (2022) Early surgery and definitive cure in small sporadic vestibular schwannoma. Acta Otorhinolaryngol Ital 42(5):481–486. 10.14639/0392-100X-N2322PMID: 36541386; PMCID: PMC9793146 36541386 10.14639/0392-100X-N2322 PMC9793146

[9] Sass HCR, West N, Møller MN et al (2018) Workup and treatment of vestibular schwannomas. Ugeskr Laeger 180(37):V02180131 30259833

[10] Batuecas-Caletrio A, Santacruz-Ruiz S, Muñoz-Herrera A et al (2013) Vestibular compensation after vestibular schwannoma surgery: normalization of the subjective visual vertical and disability. Acta Otolaryngol 133:475–480. 10.3109/00016489.2012.757798 10.3109/00016489.2012.757798 23317346

[11] Paige GD (1985) Caloric responses after horizontal canal inactivation. Acta Otolaryngol 100(5–6):321–327. 10.3109/00016488509126555 4082971 10.3109/00016488509126555

[12] Aalling M, Skals RK, Abrahamsen ER et al (2020) Comparison of test results from two separate video head impulse test systems in a cohort of patients diagnosed with a unilateral vestibular schwannoma. Eur Arch Otorhinolaryngol 277:3185–3193. 10.1007/s00405-020-06116-2 32564123 10.1007/s00405-020-06116-2

[13] Rosengren SM, Colebatch JG, Young AS et al (2019) Vestibular evoked myogenic potentials in practice: methods, pitfalls and clinical applications. Clin Neurophysiol Pract 4:47–68. 10.1016/j.cnp.2019.01.005 30949613 10.1016/j.cnp.2019.01.005 PMC6430081

[14] Taylor RL, Kong J, Flanagan S et al (2015) Prevalence of vestibular dysfunction in patients with vestibular schwannoma using video head-impulses and vestibular-evoked potentials. J Neurol 262:1228–1237. 10.1007/s00415-015-7697-4 25794859 10.1007/s00415-015-7697-4

[15] Andersen JF, Nilsen KS, Vassbotn FS et al (2015) Predictors of vertigo in patients with untreated vestibular schwannoma. Otol Neurotol 36(4):647–652. 10.1097/MAO.0000000000000668 25415462 10.1097/MAO.0000000000000668

[16] Nilsen KS, Nordahl SHG, Berge JE et al (2023) Vestibular tests related to tumor volume in 137 patients with small to medium-sized vestibular schwannoma. Otolaryngol Head Neck Surg 169:1268–1275. 10.1002/ohn.399 37337472 10.1002/ohn.399

[17] West N, Sass H, Klokker M et al (2020) Video head impulse test results in patients with a vestibular schwannoma-sensitivity and correlation with other vestibular system function tests, hearing acuity, and tumor size. Otol Neurotol 41(5):e623–e629. 10.1097/MAO.0000000000002600 32118807 10.1097/MAO.0000000000002600

[18] R Core Team (2024) _R: a language and environment for statistical computing_. R foundation for statistical computing, Vienna, Austria. https://www.R-project.org/

[19] Gouveris H, Helling K, Victor A et al (2007) Comparison of electronystagmography results with dynamic posturography findings in patients with vestibular schwannoma. Acta Otolaryngol 127:839–842. 10.1080/00016480601075357 17762995 10.1080/00016480601075357

[20] Lloyd SK, Baguley DM, Butler K et al (2009) Bruns’ nystagmus in patients with vestibular schwannoma. Otol Neurotol 30:625–628. 10.1097/MAO.0b013e3181a32bec 19471169 10.1097/MAO.0b013e3181a32bec

[21] Stipkovits EM, Van Dijk JE, Graamans K (1999) Electronystagmographic changes in patients with unilateral vestibular schwannomas in relation to tumor progression and central compensation. Eur Arch Otorhinolaryngol 256:173–176. 10.1007/s004050050135 10337507 10.1007/s004050050135

[22] Brown CS, Peskoe SB, Risoli T Jr. (2019) Associations of video head impulse test and caloric testing among patients with vestibular schwannoma. Otolaryngol Head Neck Surg 161:324–329. 10.1177/0194599819837244 10.1177/0194599819837244 30909803

[23] Hentschel M, Scholte M, Steens S et al (2017) The diagnostic accuracy of non-imaging screening protocols for vestibular schwannoma in patients with asymmetrical hearing loss and/or unilateral audiovestibular dysfunction: a diagnostic review and meta-analysis. Clin Otolaryngol 42:815–823. 10.1111/coa.12788 27905190 10.1111/coa.12788

[24] Blödow A, Helbig R, Wichmann N et al (2013) Video head impulse test or caloric irrigation? Contemporary diagnostic tests for vestibular schwannoma. HNO 61:781–785. 10.1007/s00106-013-2752-x 23959391 10.1007/s00106-013-2752-x

[25] Jensen MK, Hougaard DD (2022) Suppression head impulse testing is recommended for vestibular testing of patients with untreated unilateral vestibular schwannoma. Eur Arch Otorhinolaryngol 279:91–99. 10.1007/s00405-021-06621-y 33502546 10.1007/s00405-021-06621-y

[26] Fujiwara K, Morita S, Hoshino K et al (2024) Evaluation of semicircular Canal function using video head impulse test in patients with peripheral vestibular disorders without nystagmus. Cureus 16(6):e62786. 10.7759/cureus.62786 39036179 10.7759/cureus.62786 PMC11260217

[27] Oussou G, Magnani C, Bargiotas I et al (2022) A new sensitive test using virtual reality and foam to probe postural control in vestibular patients: the unilateral schwannoma model. Front Neurol 13:1–13 10.3389/fneur.2022.891232 PMC9174985 35693011

[28] Martin-Sanz E, Esteban-Sánchez J, González-Márquez R et al (2021) Vibration-induced nystagmus and head impulse test screening for vestibular schwannoma. Acta Otolaryngol 141:340–347. 10.1080/00016489.2021.1872797 33583327 10.1080/00016489.2021.1872797

[29] Fujiwara K, Yanagi H, Morita S et al (2019) Evaluation of vertical semicircular canal function in patients with vestibular schwannoma. Ann Otol Rhinol Laryngol 128:113–120. 10.1177/0003489418808545 30360640 10.1177/0003489418808545

[30] Abrahamsen ER, Christensen AE, Hougaard DD (2018) Intra- and interexaminer variability of two separate video head impulse test systems assessing all six semicircular canals. Otol Neurotol 39(2):e113–e122. 10.1097/MAO.0000000000001665 29315187 10.1097/MAO.0000000000001665

[31] Curthoys IS (2000) Vestibular compensation and substitution. Curr Opin Neurol 13(1):27–30 10719646 10.1097/00019052-200002000-00006

[32] Frère J, Hoffmann CP, Gauchard GC, Parietti-Winkler C (2017) Does the postural variable affect the determination of balance compensation level in vestibular schwannoma patients? Med Eng Phys 47:214–217. 10.1016/j.medengphy.2017.06.021 28687471 10.1016/j.medengphy.2017.06.021

[33] Nilsen KS, Lund-Johansen M, Nordahl SHG et al (2019) Long-term effects of conservative management of vestibular schwannoma on dizziness, balance, and caloric function. Otolaryngol Head Neck Surg 161:846–851. 10.1177/0194599819860831 31310582 10.1177/0194599819860831

[34] Murofushi T, Matsuzaki M, Mizuno M (1998) Vestibular evoked myogenic potentials in patients with acoustic neuromas. Arch Otolaryngol Head Neck Surg 124(5):509–512 9604975 10.1001/archotol.124.5.509

[35] Beyea JA, Zeitouni AG (2010) Vestibular evoked myogenic potential latencies in Meniere disease and vestibular schwannoma. J Otolaryngol Head Neck Surg 39(3):253–258 20470669

[36] Takeichi N, Sakamoto T, Fukuda S et al (2001) Vestibular evoked myogenic potential (VEMP) in patients with acoustic neuromas. Auris Nasus Larynx 28(Suppl):S39–S41. 10.1016/s0385-8146(01)00075-x 11683341 10.1016/s0385-8146(01)00075-x

[37] Ushio M, Iwasaki S, Murofushi T et al (2009) The diagnostic value of vestibular-evoked myogenic potential in patients with vestibular schwannoma. Clin Neurophysiol 120:1149–1153. 10.1016/j.clinph.2009.01.017 19394267 10.1016/j.clinph.2009.01.017

[38] Chiarovano E, Darlington C, Vidal PP et al (2014) The role of cervical and ocular vestibular evoked myogenic potentials in the assessment of patients with vestibular schwannomas. PLoS ONE 9(8):e105026. 10.1371/journal.pone.0105026 25137289 10.1371/journal.pone.0105026 PMC4138161

[39] Fujiwara K, Morita S, Fukuda A et al (2020) Analysis of semicircular canal function as evaluated by video head impulse test in patients with vestibular schwannoma. J Vestib Res 30:101–108. 10.3233/VES-200695 32200369 10.3233/VES-200695

[40] Wagner JN, Glaser M, Wowra B et al (2011) Vestibular function and quality of life in vestibular schwannoma: does size matter? Front Neurol 2:55. 10.3389/fneur.2011.00055 21941519 10.3389/fneur.2011.00055 PMC3171060

[41] Dilwali S, Landegger LD, Soares VY et al (2015) Secreted factors from human vestibular schwannomas can cause cochlear damage. Sci Rep 5:18599. 10.1038/srep18599 26690506 10.1038/srep18599 PMC4686978

[42] Huo Z, Chen J, Wang Z et al (2019) Prognostic factors of long-term hearing preservation in small and medium-sized vestibular schwannomas after microsurgery. Otol Neurotol 40:957–964. 10.1097/MAO.0000000000002284 31058754 10.1097/MAO.0000000000002284

[43] Jacob A, Robinson LL Jr, Bortman JS et al (2007) Nerve of origin, tumor size, hearing preservation, and facial nerve outcomes in 359 vestibular schwannoma resections at a tertiary care academic center. Laryngoscope 117:2087–2092. 10.1097/MLG.0b013e3181453a07 10.1097/MLG.0b013e3181453a07 17921903

